# Einflussfaktoren auf die Nutzungsintention von Pflegekräften zur Verwendung digitaler Technologien in der ambulanten Pflege – Fallstudie zur Einführung eines Sensortextils

**DOI:** 10.1007/s41449-021-00277-4

**Published:** 2021-10-13

**Authors:** Sarah Ranjana Güsken, Katrin Frings, Faizan Zafar, Timur Saltan, Paul Fuchs-Frohnhofen, Jan Bitter-Krahe

**Affiliations:** 1 Cybernetics Lab IMA & IfU, RWTH Aachen University, Dennewarstr. 27, 52068 Aachen, Deutschland; 2 Institut für Psychologie, RWTH Aachen University, Jägerstr. 17–19, 52056 Aachen, Deutschland; 3 MA&T Sell & Partner GmbH, Karl-Carstens-Straße 1, 52146 Würselen, Deutschland

**Keywords:** Nutzungsintention, Technikakzeptanz, Digitalisierung, Ambulante Pflege, Partizipative Technikentwicklung, Intention to Use, Technology Acceptance, Digitalization, Outpatient Care, Participatory Technology Development

## Abstract

Durch die steigende Arbeitsbelastung in der Pflegebranche werden Arbeitsprozesse zunehmend digitalisiert. Die Folge eines unzureichenden Einbezugs von Pflegekräften in diesen Digitalisierungsprozess zeigt sich in schlecht integrierten technologischen Entwicklungen mit ausbleibender Technikakzeptanz. Um eine Basis für eine erfolgreiche Technikentwicklung in der ambulanten Pflege legen zu können, gilt es zunächst die Nutzungsabsicht ambulanter Pflegekräfte besser zu verstehen. Zur Untersuchung der Nutzungsabsicht wird in dieser Arbeit ein Modell entwickelt, das Einflussfaktoren auf die Nutzungsintention von digitalen Technologien in der ambulanten Pflege anhand einer Fallstudie zur Einführung einer textilen Sensormatte aufzeigt. Im entwickelten Modell wird erstmals neben der Technikakzeptanz auch die Pflegesituation als Einflussfaktor auf die Nutzungsintention betrachtet und untersucht. Neben der Herleitung der Faktoren des Modells und der Untersuchung der Stärke ihrer Einflüsse wird die praktische Relevanz für Technikentwickler*innen auch in anderen Pflegekontexten abgeleitet.

*Praktische Relevanz*: Der demographische Wandel in der deutschen Bevölkerung führt zu großen Herausforderungen in unterschiedlichen Branchen. In der ohnehin bereits stark vom Fachkräftemangel betroffenen Pflegebranche macht sich dieser Wandel besonders in einer Zunahme pflegebedürftiger Menschen in der ambulanten Pflege und einer hohen Arbeitsbelastung der Beschäftigten bemerkbar. Zur Reduktion dieser Arbeitsbelastung werden daher vermehrt digitale Technologien verwendet, die den Arbeitsalltag von Pflegekräften hinsichtlich physischer und psychischer Faktoren erleichtern sollen. Das in dieser Studie entwickelte Modell beschreibt – auf Basis eines Fallbeispiels zur Einführung einer Sensormatte – förderliche und hinderliche Faktoren für die Technikeinführung in der ambulanten Pflege und trägt so zu einer gelingenden Digitalisierung in diesem Berufsbereich bei.

## Abstract (en)

Operational processes in the care sector are becoming increasingly digitalized due to rising workload. Insufficient involvement of caregivers in this digitalization process results in poorly integrated technological developments along with a lack of technology acceptance. Therefore, in order to lay a firm foundation for successful technology development, it is utmost essential to understand the intention of professionals to use technology in outpatient care. This paper develops a model that outlines the factors influencing the intention of caregivers to use digital technologies in outpatient care, based on a case study examining the introduction of a textile sensor mat. In the developed model, the care situation is investigated for the first time as a factor influencing the intention to use digital technologies, in addition to technology acceptance. Apart from deriving the factors of the model and inspecting the strength of their influences, the practical relevance for technology developers in other care contexts is also determined.

*Practical Relevance*: Demographic change in the German population is leading to major challenges in various sectors. Specifically, in the outpatient care sector, which is already severely affected by a shortage of skilled workers, this change is particularly noticeable with an increase in patients and a high workload for caregivers. Therefore, digital technologies are increasingly being used to make the daily work of caregivers easier in terms of physical and psychological factors. The model developed in this study describes—based on a case study on the introduction of a textile sensor mat—conducive and obstructive factors for the introduction of technology in outpatient care and thus contributes to successful digitalization in this field.

## Einleitung

Die Pflegebranche steht angesichts der Zunahme pflegebedürftiger Menschen, eines überdurchschnittlichen Krankenstandes der Beschäftigten und eines wachsenden Fachkräftemangels vor besonderen Herausforderungen (Kliner et al. 2017). Nach Vorausberechnungen des Statistischen Bundesamtes stehen in Deutschland im Jahr 2060 100 Personen im Alter von 20 bis 60 Jahren 82 Personen die älter als 60 Jahre sind gegenüber, während das Verhältnis 2015 noch 100:50 war (Statistisches Bundesamt 2017). Viele Einrichtungen und ambulante Diensten stehen deshalb schon heute vor der Herausforderung, dass nicht genügend Personal gefunden werden kann, um die große Nachfrage nach Pflegedienstleistungen zu befriedigen. Bei einer Fortsetzung dieser aktuellen Entwicklungen werden im Jahr 2030 fast 500.000 Vollzeitkräfte in der Pflege fehlen (Deutscher Pflegerat 2020). Hinzu kommt, dass die finanzierbaren Stellenbesetzungen in der ambulanten und stationären Pflege, selbst wenn der Arbeitsmarkt genügend Bewerber*innen anbietet, von vielen Beschäftigten als weitgehend unzureichend und unattraktiv bezeichnet werden. Seit vielen Jahren wird darüber geklagt, dass körperliche wie psychische Belastungen (Böhle und Glaser 2006) der professionell Pflegenden zu einer verminderten Arbeitszufriedenheit (Fuchs-Frohnhofen et al. 2010) führen und den Beschäftigungsverbleib stark gefährden (Pohl 2011). Die gesellschaftliche Zielsetzung, diesen Bereich zu entlasten, durch beispielsweise bessere Anreizmodelle für den Pflegeberuf, wird deshalb schon seit Jahren verfolgt, jedoch mit eher mäßigem Erfolg wie aus dem sich verschärfenden Fachkräftemangel erkennbar ist (Fachinger 2017).

Mit voranschreitender Digitalisierung hat sich der Pflegebereich wirtschaftlich zu einem attraktiven Markt entwickelt (Compagna 2018). Allerdings haben sich unterstützende Systeme in der Pflege bisher aufgrund mangelnder Anwender*innenakzeptanz noch nicht flächendeckend durchsetzen können. Um im Pflegebereich zu Verbesserungen zu kommen, ist deshalb eine Auseinandersetzung mit den spezifischen Anforderungen der Profession „Pflege“ an die Technikentwicklung als Arbeitsunterstützung wesentlich, um die berufsspezifischen Bedarfe adäquater zu adressieren. Ebenfalls wird hierdurch die Gefahr vermindert, Lösungsangebote bereitzustellen, die den Beschäftigten dieser Branche nicht gerecht werden (können).

### Spezifika des Pflegeberufs

Pflege als professionelle Dienstleistung umfasst alle Aspekte der präventiven, rehabilitativen, kurativen, palliativen und kompensatorischen Versorgung von Menschen mit bestehenden oder zu erwartenden Hilfebedarfen (Ströbel und Weidner 2003). Dabei geht es bei beruflicher Pflegearbeit um die Begleitung und Unterstützung von Menschen bei deren individueller Lebensgestaltung unter besonderer Berücksichtigung gesundheitsbezogener Fragestellungen und der Bearbeitung bzw. Integration gesundheitsbezogener Einschränkungen. Dazu gehört, Unterstützungsbedarfe zu ermitteln und Unterstützung im Rahmen umfassender Versorgungskonzepte zu organisieren oder selbst durchzuführen.

Im Rahmen der Konzertierten Aktion Pflege (KAP: Die Bundesregierung 2020) wird auf verschiedenen Ebenen daran gearbeitet, die Arbeitssituation der Pflegekräfte zu verbessern, zumal deren Bedeutung durch die aktuelle SARS-CoV-2-Pandemie im gesellschaftlichen Bewusstsein gestiegen ist. In der Arbeitsgruppe „Innovative Versorgungsansätze und Digitalisierung“ werden u. a. verschiedene Projekte initiiert, die dazu beitragen sollen, digitale Technologien in der ambulanten und stationären Pflege so zu entwickeln und einzuführen, dass sie einen Beitrag dazu leisten, Belastungen für die Pflegekräfte durch ihre Arbeit zu reduzieren. Dabei wird auch an Diskussionen angeknüpft, die im Rahmen verschiedener Modellprojekte des BMBF gefordert haben, dass neue Technologien in der Pflege so beschaffen sein müssen, dass sie die Bedarfe und Bedürfnisse der Nutzer*innen erfüllen und bezahlbar in der Breite der Branche zur Verfügung stehen. Weinberger stellt hierzu fest, dass zwar viele Systeme am Markt verfügbar sind, aber *„(…) trotz der Marktverfügbarkeit und der durch positive Evaluierung in Feldtests ausgewiesenen Potenziale wird bisher der Markt nicht durchdrungen, d.* *h., die Produkte kommen bis auf wenige Ausnahmen nicht im Pflegealltag an (…)“ *(Weinberger und Decker 2015, S. 37).

Deswegen fordern die Autor*innen des Memorandums „Arbeit und Technik 4.0 in der professionellen Pflege“ auch bei der Entwicklung und Einführung neuer pflegeunterstützender Technologien

- den Kommunikationsprozess zwischen Pflegebedürftigen und Pflegenden in der Interaktionsarbeit Pflege (Böhle et al. 2014) nicht aus dem Blick zu verlieren,
- relevante Akteur*innen aus der Pflege zu beteiligen,
- und die arbeitsentlastende Einbindung neuer Technologien in pflegerische Arbeits- und Organisationsprozesse rechtzeitig und ausreichend zu berücksichtigen (Fuchs-Frohnhofen et al. 2018, S. 3 ff.).

Zusammenfassend werden durch die fortschreitende Digitalisierung tiefgreifende Veränderungen im Pflegeberuf erwartet – insbesondere in Bezug auf die Unterstützung und Entlastung der Pflegenden. Die erfolgreiche Einführung digitaler Technologien ist aber immer auch von der Akzeptanz und Nutzungsabsicht der Nutzenden abhängig. In Bezug auf den Pflegeberuf existieren hierfür jedoch bislang wenige aussagekräftige Studien und Fallbeispiele. Derartige Untersuchungen sind notwendig, um zu einer gezielten und innovativen Entwicklung und Einführung digitaler Pflegetechnologien beizutragen und damit die erwarteten Veränderungen positiv für den Pflegeberuf und die Pflegenden zu gestalten (Raehlmann 2017; Rohpol und Weyer 2013).

### Die Untersuchung von Nutzungsintention einer digitalen Pflegetechnologie anhand eines Fallbeispiels

Die Untersuchung der Nutzungsintention von Pflegetechnologie unter Einbezug des Pflegepersonals ist intendiertes Ziel des Forschungsprojekts DigiKomp-Ambulant. In diesem wird anhand des Fallbeispiels einer textilen Sensormatte ein partizipativer Technikentwicklungsprozess gestaltet und jene Faktoren untersucht, die auf die Absicht der Nutzung digitaler Technologie von Arbeitskräften in der ambulanten Pflege wirken. Die im Fallbeispiel zum Einsatz kommende Sensormatte überwacht Bewegungs- und Vitalwerte und sendet die digitalen Daten an ein durch die Pflegekraft gesteuertes internetfähiges digitales Endgerät. Die im Rahmen dieses Projekts gewonnenen Daten dienen als Fallbeispiel für den vorliegenden Beitrag und bilden somit die Basis für die durchgeführte Studie und deren Ergebnisse.

Ziel dieses Beitrags ist es Einflussfaktoren und ihre Einflussstärke auf die Nutzungsintention von Pflegekräften von Technologie in der ambulanten Pflege aufzuzeigen. Hierfür wird zunächst eine theoretische Grundlage gelegt (Absatz 2) und schließlich ein fallbeispielbezogenes pflegespezifisches Akzeptanzmodell aufgestellt (Absatz 3 und 4). Zur Herausarbeitung der Einflussstärke wird das Modell vor Einführung einer digitalen Pflegetechnologie im Rahmen eines Fallbeispiels durch einen Online-Fragebogen getestet (Absatz 5). Das Modell soll schließlich zum besseren Verständnis der Technikakzeptanz in der ambulanten Pflege beitragen und die Faktoren aufzeigen, die eine Technologienutzung in der ambulanten Pflege maßgeblich beeinflussen können (Absatz 5 und 6).

## Theoretische Fundierung

### Technologieakzeptanzmodelle

**Figure 1:**
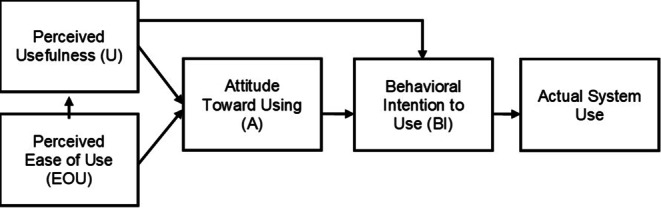

Die Erforschung von Nutzungsintentionen neuer Technik und der daraus folgenden Technikakzeptanz erfolgt durch die Aufstellung und Überprüfung von sogenannten Technikakzeptanzmodellen. Den meisten dieser allgemeinen Modelle liegt die *Theory of Reasoned Action* (TRA, Theorie des überlegten Handelns) von Fishbein und Ajzen (1975) zugrunde, welche die Annahme postuliert, dass menschliches Verhalten von persönlichen Einstellungen bestimmt ist. Davis, Bagozzi und Warshaw haben 1989 die TRA, damals explizit auf die Computernutzung im Arbeitskontext, übertragen und das weitverbreitete Technikakzeptanzmodell (TAM) aufgestellt (Davis et al. 1989). Das TAM beschreibt drei einstellungsbildende Hauptfaktoren zur Vorhersage des tatsächlichen Nutzungsverhaltens: *Perceived Usefulness* (U; wahrgenommene Nützlichkeit), *Perceived Ease of Use* (EOU; wahrgenommene Einfachheit der Nutzung) sowie *Attitude Towards Using* (A; Einstellung zur Nutzung). Dabei wirken sich die *Perceived Usefulness* und der *Perceived Ease of Use* auf die *Attitude Towards Using* aus, welche wiederum gemeinsam mit der *Perceived Usefulness* die *Behavioral Intention to Use* (BI; Nutzungsabsicht) voraussagt. Der tatsächliche Gebrauch der Technologie (*Actual System Use*) resultiert am Ende der Kette direkt aus der Behavioral Intention to Use (vgl. Abb. 1).

Das ursprüngliche TAM-Modell wurde im Jahr 2000 von Venkatesh und Davis durch soziale Einflüsse, wie z. B. subjektive Normvorstellungen, Freiwilligkeit der Nutzung und Imageerzeugung, ergänzt und somit zum TAM 2 weiterentwickelt. Darauffolgend integrierten Venkatesh et al. (2003) acht verschiedene Technikakzeptanzmodelle zu vier erklärenden Hauptfaktoren für das Außmaß einer Techniknutzung in der *Unified Theory of Acceptance and Use of Technology *(UTAUT). Das beschreibende UTAUT-Modell zeigt, dass Techniknutzung durch die *Performance Expectancy* (Leistungserwartung), *Effort Expectancy* (Annahmen über das Ausmaß an Aufwand, mit welcher die Verwendung verbunden ist), *Social Influence* (soziale Einflüsse) und *Facilitating Conditions* (Rahmenbedingungen die die Verwendung der Technologie erleichtern) vorhergesagt werden können.

Lösgelöst vom UTAUT-Modell entwickelten Venkatesh und Bala (2008) das TAM 1 und TAM 2 zu einem neuen Modell, dem TAM 3, weiter. Im TAM 3 liegen die vier Gruppen von Faktoren für die Erklärung von Techniknutzung wieder näher am ursprünglichen Basismodell und umfassen *Individual Differences* (Individuelle Unterschiede zwischen Personen), *Social Influence* (soziale Einflussfaktoren), *System Characteristics* (technologiespezifische Charakteristika) und *Facilitating Conditions* (Rahmenbedingungen der Nutzung). Mit über 20 Studien (z. B. Ahmed et al. 2020; Barzegari et al. 2020; Dünnebeil et al. 2012; Holden und Karsh 2010; Nguyen et al. 2020; Vadillo et al. 2017; Zöllick et al. 2020), die die verschiedenen Entwicklungsversionen des TAMs im Gesundheitswesen bereits gestestet haben, wird sie zunehmend als geeignete Theorie für die Übertragung auf den Gesundheitskontext angenommen (Holden und Valdez 2019).

**Figure 2:**
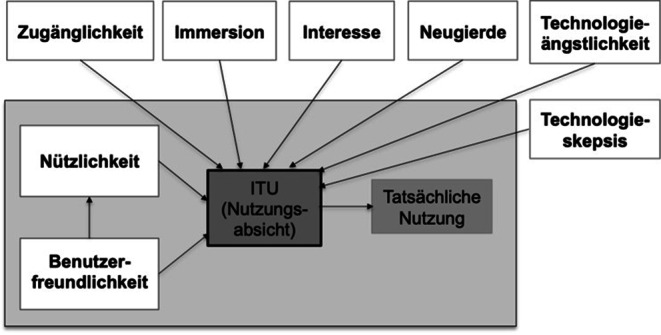

Da bei den Technologieakzeptanzmodellen (TAM 1–3) wichtige psychologische Konstrukte, wie z. B. kognitive Fähigkeiten und Selbstwirksamkeit (Czaja et al. 2006) oder Vorerfahrungen mit Technik (Arning und Ziefle 2007) nicht hinlänglich berücksichtigt werden, konstruierten Kothgassner et al. (2012) das *Technology Usage Inventory* (TUI) (Abb. 2). Neben der Integration psychologischer Einflussfaktoren gestalteten sie das Inventar unabhängig von der Art der zu nutzenden Technologie und ermöglichen dadurch die Evaluation neu entwickelter oder zu entwickelnder Technologien. Die vielfältige Anwendungsbreite des TUIs wurde durch Forschung in diversen Technikakzeptanzthemen gezeigt, z. B. bei der Untersuchung der Nutzungsabsicht von elektronischen Schulbüchern (Froitzheim et al. 2017) oder der Erforschung von Gamingverhalten junger Frauen (Felnhofer et al. 2013). Durch eine einfache und leicht verständliche Item-Charakteristik kann der TUI für eine breite und heterogene Zielgruppe verwendet werden.

### Technikakzeptanz in der Pflege

Bisherige Literatur zur Technikakzeptanz im Pflegekontext kann in zwei übergeordnete Forschungsfelder unterschieden werden (Fachinger 2017): (a) *Technische Assistenzsysteme für den Pflegeprozess* und (b) *Ambient Assisted Living* (AAL).

Im Forschungsfeld (a) geht es insbesondere um die Frage, wie der Einsatz von Technik den Arbeitsalltag von stationären Pflegekräften unterstützen kann (Hülsken-Giesler und Remmers 2020; Kuhlmey et al. 2019). Im Forschungsfeld (b) stehen technische Hilfsprodukte in der ambulanten Pflege im Fokus. Hier werden Produkte und der Produktumgang mit Technik betrachtet, die pflegebedürftigen Menschen das selbstständige Leben zuhause länger ermöglichen sollen (Fachinger et al. 2012; Hülsken-Giesler und Remmers 2020; Rayan et al. 2021; Scorna et al. 2021).

Die pflegespezifische Technikakzeptanzforschung konzentriert sich im Forschungsbereich (a) stark auf den Einsatz von Informations- und Kommunikationstechnologien, die beispielsweise digitalisierte Pflegedokumentationen, telemedizinische Leistungen sowie die Steuerung von Arbeits- und Organisationsprozessen ermöglichen oder erleichtern sollen (Hielscher et al. 2017; Urban und Schulz 2020; Vadillo et al. 2017). Studien zur Evaluation der Nutzung dieser Technologien finden fast ausschließlich im stationären Bereich statt und bilden die Akzeptanz der Pflegekräfte meist in Abhängigkeit von persönlichen Eigenschaften und professionsunspezifisch wie beispielsweise Geschlecht, Alter, Berufserfahrung Bildungsniveau und sozialen Verhältnissen ab (Kuhlmey et al. 2019; Zöllick et al. 2020; Hülsken-Giesler et al. 2019). Van Heek et al. (2018) zeigen z. B. mit einem Regressionsmodell auf der Basis eines Datensatzes von 287 Befragten, dass stationäre Pflegekräfte professionsspezifisch deutlich kritischer dem Einsatz von technologischen Hilfsmitteln in ihrem Arbeitsalltag gegenüberstehen als andere Berufsgruppen. Hieraus leiten Evans et al. (2018) ab, dass der Grund für ausbleibenden Technikeinsatz in der Pflege weniger an einer Technikaversion der Pflegekräfte liegt, sondern an einer mangelnden Beachtung der pflegespezifischen Ansprüche an die Technikgestaltung.

Klassische AAL-Technologien adressieren mit den Pflegebedürftigen selbst eine andere Zielgruppe als technische Assistenzsysteme für den Pflegeprozess. Die Akzeptanz der Zielgruppe der Pflegebedürftigen wurde im Forschungsbereich (b) in einigen Studien untersucht. In diesen wurden Faktoren wie Alter, kognitive Fähigkeiten, Aussehen der Technik, Sicherheit und Vertrauenswürdigkeit als Einflussfaktoren identifiziert (Choukou et al. 2021; van Heek und Ziefle 2018; Marschollek et al. 2014; Spinsante et al. 2017; Offermann-van Heek und Ziefle 2018). Die Erforschung der Technikakzeptanz zum Einsatz von AAL-Technologien bei ambulantem Pflegepersonal steckt derzeit allerdings noch in den Kinderschuhen (Zöllick et al. 2020; Choukou et al. 2021).

Aufgrund der unterschiedlichen Technologien, Zielgruppen und Pflegesituationen der beiden vorgestellten Forschungsbereiche sind die Faktoren der Technikakzeptanz von Pflegepersonal nur bedingt von der Untersuchung des Bereichs (a) auf den Bereich (b) übertragbar und indizieren so eine gesonderte Untersuchung (Broadbent et al. 2009; Choukou et al. 2021; Savela et al. 2018; Zöllick et al. 2020). Gemeinsam haben die Forschungsfelder auf einer übergeordneten Ebene jedoch, dass die Technikakzeptanz und Nutzungsabsicht stets in Verbindung mit der konkreten Pflegesituation zu bewerten ist, da diese die Arbeit ambulanter Pflege maßgeblich charakterisiert (Bleses und Busse 2020). Die Ursachen für eine mangelnde Durchsetzungskraft neuer Technologien sind darüber hinaus jedoch vielfältig (Singh et al. 2017). Der Hauptgrund liegt, äquivalent zum Forschungsfeld der Technologien für Pflegekräfte, in einer nicht ausreichend zielgruppengerechten Berücksichtigung der spezifischen Bedarfe, Erwartungen sowie Kompetenzen von Pflegekräften (Spinsante et al. 2017; Fuchs-Frohnhofen et al. 2020).

## Herleitung Forschungsmodell und Hypothesen

Um Einflussfaktoren und ihre Einflussstärke auf die Nutzungsintention von Pflegekräften von Technologie in der ambulanten Pflege aufzuzeigen wurde ein fallbeispielbezogenes pflegespezifisches Akzeptanzmodell aufgestellt. Übergeordnetes Ziel ist es eine Modellgrundlage herzuleiten, mit deren Hilfe Nutzungsabsichten neuer Technologien im Kontext ambulanter Pflege besser verstanden werden können.

Die Herleitung des Modells erfolgte in vier Schritten. **(1)** Literaturanalyse (siehe Abschn. 2), **(2)** Übertragung klassischer Technologieakzeptanzfaktoren auf den konkreten ambulanten Pflegekontext, **(3)** qualitative Workshops zum Einbezug der konkreten Pflegesituation und **(4)** Hypothesenformulierung.

### Übertragung klassischer Technologieakzeptanzfaktoren auf den konkreten ambulanten Pflegekontext

Zur Beschreibung der Technologieakzeptanz wurde das von Kothgassner et al. (2012) entwickelte *Technology Usage Inventory* (TUI) als das Grundlagenmodell mit der höchsten Passung zur Erforschung der Technikakzeptanz in der Pflege erachtet. Der technologieunabhängige TUI basiert auf dem TAM und ist als eine Erweiterung des Technologieakzeptanzmodells zu verstehen. Der TUI ergänzt die klassischen Technologieakzeptanzfaktoren des TAM, wie z. B. Perceived Usability, Peceived Ease of Use, Technology Anxiety etc. mit wichtigen psychologischen Konstrukten, wie z. B. Alltagsbewältigung und Emotionale Vertrautheit. Diese psychologischen Konstrukte spielen insbesondere in der Interaktion zwischen Pflegekraft und zu pflegender Person eine elementare Rolle und sind deshalb in der Modellkonstruktion miteinzubeziehen.

Für den Modellstrang der Technikakzeptanz wurden die den sieben Faktoren des TUIs (Neugierde, Angst, Interesse, Skepsis, Benutzerfreundlichkeit, Nützlichkeit und Zugänglichkeit sowie Nutzungsintention) zugehörigen 34 Items an das Setting des Fallbeispiels angepasst und operationalisiert (siehe Tab. 1).

**Table 1:** 

Nr.	Item	Faktorzugehörigkeit der Items im Modellstrang Technikakzeptanz
1	Ich bin neugierig auf den Einsatz von Technik in der (ambulanten) Pflege	Neugierde
2	Ich mache mir oft Sorgen darüber, dass mich neue technische Geräte überfordern könnten	Angst
3	Ich wollte mich schon früher mit dem Einsatz von Technik in der (ambulanten) Pflege beschäftigen	Neugierde
4	Wenn ich ein neues technisches Gerät verwenden soll, bin ich erst mal misstrauisch	Angst
5	Ich bin bestrebt mehr über den Einsatz von Technik in der (ambulanten) Pflege zu erfahren	Neugierde
6	Mir fällt es schwer technischen Geräten zu vertrauen	Angst
7	Mich hat die Verwendung von Technik in der Pflege schon immer interessiert	Neugierde
8	Die Vorstellung, bei der Verwendung technischer Geräte etwas falsch zu machen, macht mir Angst	Angst
9	Im Laufe meines Lebens habe ich mir viel technisches Wissen angeeignet	Interesse
10	Ich versuche stets aktuelle Informationen über neue technische Entwicklungen in der Pflege zu bekommen	Interesse
11	Ich erwarte, dass die Anwendung der textilen Sensormatte die ambulante Pflegesituation für mich als Pflegekraft komfortabler macht	Nützlichkeit
12	Ich denke, dass die Nutzung der textilen Sensormatte mit einem gewissen Risiko verbunden ist	Skepsis
13	Ich erwarte, dass die Anwendung der textilen Sensormatte leicht verständlich ist	Benutzerfreundlichkeit
14	Wenn ein neues technisches Gerät für die Pflege auf den Markt kommt, informiere ich mich darüber	Interesse
15	Wenn ein neues technisches Gerät für die Pflege in meiner Pflegeeinrichtung angeschafft wird, informiere ich mich darüber	Interesse
16	Ich denke, dass die textile Sensormatte mir hilft, meine täglichen Aufgaben in der ambulanten Pflege bequemer zu erledigen	Nützlichkeit
17	Ich denke, dass die textile Sensormatte Gefahren für mich birgt	Skepsis
18	Ich denke, dass die Anwendung der textilen Sensormatte insgesamt einfach ist	Benutzerfreundlichkeit
19	Ich denke, dass sich die textile Sensormatte finanziell fast jeder Pflegedienst leisten könnte	Zugänglichkeit
20	Ich denke, dass sich die textile Sensormatte finanziell alle zu pflegenden Personen leisten könnten	Zugänglichkeit
21	Auf Basis der mir vorliegenden Informationen würde ich meinen zu pflegenden Personen empfehlen, sich die textile Sensormatte anzuschaffen	Nützlichkeit
22	Ich denke, dass die Sensormatte meinen Arbeitsalltag stört	Skepsis
23	Ich denke, dass die Anwendung der textilen Sensormatte kompliziert ist	Benutzerfreundlichkeit
24	Ich denke, dass die textile Sensormatte grundsätzlich für jeden erhältlich ist	Zugänglichkeit
25	Ich denke, dass die Sensormatte nicht für jede zu pflegende Person verwendbar ist	Zugänglichkeit
26	Ich denke, dass die textile Sensormatte mich dabei unterstützt, meine alltäglichen Aufgaben in der ambulanten Pflege zu erfüllen	Nützlichkeit
27	Ich denke, dass die Anwendung der textilen Sensormatte mir mehr Nachteile als Vorteile bringt	Skepsis
28	Ich denke, dass die Anschaffung der textilen Sensormatte mit wenig Aufwand verbunden ist	Zugänglichkeit
29	Ich informiere mich über technologische Entwicklungen	Interesse
30	Ich würde die textile Sensormatte in meinem Arbeitskontext anwenden	Nutzungsintention
31	Ich würde die textile Sensormatte in meinem privaten Kontext anwenden	Nutzungsintention
32	Ich wünsche mir, dass eine textile Sensormatte in meinem Arbeitskontext der ambulanten Pflege angeschafft wird	Nutzungsintention
33	Ich hätte gerne Zugang zu einer textilen Sensormatte für den Pflegekontext	Nutzungsintention
34	Ich hätte gerne Zugang zu einer textilen Sensormatte im privaten Kontext	Nutzungsintention

### Herleitung der Faktoren zur konkreten Pflegesituation am Fallbeispiel:

Vorangegangene Forschung bezüglich des Einsatzes von Technikunterstützung in der Pflege betont, dass die Nutzungsabsicht einer assistiven Technologie über die reine Technikakzeptanz hinaus auch davon beeinflusst wird, ob sie die Qualität der aktuellen Pflegesituation signifikant verbessern kann und die pflegespezifischen Bedarfe ausreichend adressiert (Evans et al. 2018; Fuchs-Frohnhofen et al. 2020; Offermann-van Heek und Ziefle 2018). Eine Inklusion der Pflegesituation in die Untersuchungen von modellbasierter Nutzungsintention bleibt in der Forschung bisher jedoch weitestgehend aus. Um die Nutzungsintention im vorliegenden Fallbeispiel möglichst umfassend erklären zu können werden die berufsspezifischen Faktoren zur Pflegesituation im vorgeschlagenen Modell ebenfalls berücksichtig.

Zur Operationalisierung der aktuellen Pflegesituation und der erwarteten Veränderung durch den Einsatz der Sensormatte wurde zu Beginn der Untersuchung ein Workshop mit Pflegekräften von zwei im Projekt kooperierenden Pflegediensten durchgeführt. Im Rahmen des Workshops wurde zunächst die Pflegesituation und der Pflegekontext in der ambulanten Pflege mit und ohne Techniksupport definiert:

Die Pflegekräfte kommen zu den Pflegebedürftigen nach Hause und führen dort die Grundversorgung durch. In einer bisherigen Pflegesituation liegen dem Pflegepersonal bisher die Stammdaten, Diagnosen und evtl. Pflegeberichte aus den vorherigen Tagen vor. Aktuelle Vitaldaten und Bewegungsprofile müssen entweder erhoben oder durch Begutachtung des Körpers bewertet werden oder können nicht in die Pflegesituation miteinbezogen werden. Durch den Einsatz einer digitalen Technologie wie z. B. eine textile Sensormatte können die Pflegekräfte mit elektronischen Daten über die Pflegebedürftigen informiert werden. Für die Pflegesituation werden dann drei Zeiträume relevant: (1) vor der Ankunft bei der pflegebedürftigen Person, (2) während der Tätigkeit vor Ort, (3) nach Abfahrt der Pflegekraft. Die relevanten Daten könnten entweder kontinuierlich oder punktuell (z. B. unmittelbar vorher/nachher oder ausschließlich während der Pflegesituation) übermittelt bzw. abgerufen werden.

In einem anschließenden Schritt wurden gemeinsam mit den Pflegekräften Faktoren und Auswirkungen aus dem Pflegeeinsatz mit und ohne unterstützende Pflegetechnologie anhand von Berichten aus der täglichen Arbeit diskutiert und dokumentiert. Hierbei zeigte sich, dass insbesondere der Faktor Stress, also z. B. Unsicherheit was die Pflegekraft vor Ort erwartet oder Angst vor Auswirkungen falscher Dokumentation aufgrund von Zeitmangel besonders großen Raum in der Beschreibung der Pflegesituation einnimmt. Die Berichte, Anmerkungen und Diskussionen bildeten die Grundlage der Faktorenextraktion für den Modellstrang der Pflegesituation und wurden zur Operationalisierung als Faktoren mittels einer qualitativen Inhaltsanalyse aufbereitet. Hieraus wurden sechs Faktoren als Einfluss auf die ambulante Pflegesituation abgeleitet: Zeit während der Pflege, Stressempfinden, Interaktionsqualität mit den zu pflegenden Personen, Ausstattung (bezüglich verfügbarer Pflegehilfsmittel), Informationsstand über den Zustand der zu pflegenden Personen und Vorbereitung auf die häusliche Pflegesituation.

Die abgeleiteten Faktoren wurden wiederum durch jeweils vier bis elf Items beschrieben (vgl. Tab. 2). Die qualitativ erarbeiteten Faktoren und Items wurden nach ihrer Aufarbeitung durch die am Projekt teilnehmenden Pflegekräfte bezüglich ihrer Passung und Vollständigkeit validiert.

**Table 2:** 

Nr.	Item	Faktorzugehörigkeit der Items im Modellstrang Pflegesituation
*Vor Ankunft bei den zu pflegenden Personen:*		
1	Ich habe genügend Zeit mich auf die zu pflegenden Personen vorzubereiten	Zeit
2	In der aktuellen Pflegesituation liegen mir vor der Ankunft bei der zu pflegenden Person Informationen zur Bewegungsintensität seit dem letzten Besuch des Pflegedienstes vor	Vorbereitung
3	In der aktuellen Pflegesituation liegen mir vor der Ankunft bei der zu pflegenden Person Informationen zu den Vitaldaten seit dem letzten Besuch des Pflegedienstes vor	Vorbereitung
4	Ich würde mich gerne besser auf die zu pflegenden Personen vorbereiten	Vorbereitung
5	In der aktuellen Pflegesituation liegen mir vor der Ankunft bei der zu pflegenden Person Informationen zu möglicher Nässe im Bett seit dem letzten Besuch des Pflegedienstes vor	Vorbereitung
6	Im Vergleich zu meiner aktuellen Arbeitssituation: Zur besseren und individuellen Vor- und Nachbereitung der Pflegesituation würde ich mir vor meiner Ankunft mehr Daten zum Befinden der zu pflegenden Person wünschen (Puls, Atemfrequenz, Bewegungsdaten, Bettnässeinformationen)	Vorbereitung
7	In der aktuellen Pflegesituation liegen mir vor Ankunft bei der zu pflegenden Person ausreichend Informationen zu Geschehnissen seit dem letzten Besuch des Pflegedienstes vor	Vorbereitung
8	Ich mache mir manchmal Sorgen, was mich bei den zu pflegenden Personen bei der Ankunft erwartet	Stress
*Während der Tätigkeit vor Ort:*		
9	In der aktuellen Pflegesituation ist für das Messen des Pulses genügend Zeit vorgesehen	Zeit
10	In der aktuellen Pflegesituation ist für das Messen des Körpergewichts genügend Zeit vorgesehen	Zeit
11	Bei Wegfall der Messung und Dokumentation von Puls und Körpergewicht, hätte ich spürbar mehr Zeit zur Erfassung anderer relevanter Vitalparameter (wie z. B. Blutdruck, Blutzucker oder Körpertemperatur)	Zeit
12	Die Aufzeichnung der Atemfrequenz durch die textile Sensormatte halte ich für eine hilfreiche Funktion in der ambulanten Pflege	Ausstattung
13	Ich habe genügend Zeit, um mit den zu pflegenden Personen über alltägliche Dinge zu sprechen	Interaktionsqualität
14	Um mit den zu pflegenden Personen auch über die reine Pflege hinaus zu interagieren, fehlt mir häufig die Zeit	Interaktionsqualität
15	Ich habe Zeit, um mit den Angehörigen der zu pflegenden Personen in den Austausch zu treten	Interaktionsqualität
16	Ich denke, dass ich durch den Einsatz der Sensormatte mehr Zeit für die soziale Interaktion mit den Angehörigen der zu pflegenden Person habe werde	Interaktionsqualität
17	Zur Messung einzelner Vitalparameter (z. B. Körpergewicht) fehlen mir vor Ort teils Hilfsmittel oder Geräte	Ausstattung
18	Ich kann nachvollziehen, ob sich die zu pflegende Person in meiner Abwesenheit selbstständig umgelagert hat	Informationsstand
19	Zur Vorbeugung eines Dekubitus oder anderer chronischer Wunden wünsche ich mir mehr Informationen über das Ausmaß der Bewegungen der zu pflegenden Person	Informationsstand
20	Die Bewegungsintensität der zu pflegenden Person in meiner Abwesenheit wird mir häufig von den Betroffenen selbst oder von deren Angehörigen berichtet	Informationsstand
21	Die Selbstauskünfte über die Bewegungsintensität der zu pflegenden Personen erscheinen mir als unzuverlässig und verzerrt	Informationsstand
22	Zur angemessenen Thromboseprophylaxe sind mehr Informationen über das Ausmaß der Bewegungen der zu pflegenden Personen in meiner Abwesenheit hilfreich	Informationsstand
23	Zur Überwachung von gesundheitlichen Veränderungen bzw. der Prophylaxe der zu pflegenden Personen (z. B. Erkennen einer Unterversorgung, Dekubitusvermeidung) genügen die Daten, die ich derzeit ambulant während der Pflegesituation erhebe	Ausstattung
24	Bei der Dokumentation der Vitaldaten der zu pflegenden Personen habe ich Sorgen etwas zu vergessen oder falsch einzutragen	Stress
*Nach Abfahrt der Pflegekraft:*		
25	Nach der Versorgung und Abfahrt mache ich mir oft Gedanken über den Zustand der zu pflegenden Personen	Stress
26	Mir fehlt die Möglichkeit, nach Abreise in kritischen Gesundheitssituationen bestimmte Vitaldaten der zu pflegenden Personen beobachten zu können	Informationsstand
27	Die Möglichkeit zur dauerhaften Überwachung der Vitaldaten aller zu pflegenden Personen nach der Abreise würde bei mir Stress auslösen	Stress
28	Die Möglichkeit zur dauerhaften Überwachung der Vitaldaten aller zu pflegenden Personen nach der Abreise würde bei mir ein beruhigendes Gefühl auslösen	Stress
29	Die Möglichkeit zur Überwachung der Vitaldaten einzelner zu pflegenden Personen in kritischen Situationen würde bei mir Stress auslösen	Stress
30	Die Möglichkeit zur Überwachung der Vitaldaten einzelner zu pflegenden Personen in kritischen Situationen würde bei mir ein beruhigendes Gefühl auslösen	Stress
31	Ich wäre beruhigter, wenn ich wüsste, dass in bestimmten Situationen die Angehörigen oder ein Notdienst durch ein Alarmsystem benachrichtigt werden würden	Stress
32	In der aktuellen Pflegesituation verlasse ich die zu pflegenden Personen häufig mit einem unguten Gefühl	Stress
33	Stellen Sie sich bitte vor, dass Sie als Pflegekraft bei der Abfahrt entscheiden sollen, ob eine zu pflegende Person wegen eines kritischen Zustands durch die Daten der Sensormatte überwacht werden soll. Diese Entscheidung zu treffen, löst in mir Unbehagen aus	Stress
34	Ich halte die Sensormatte für ein nützliches Hilfsmittel um Inkontinenz besser diagnostizieren zu können	Ausstattung
35	Mit der mir aktuell möglichen Gesamtpflege der zu pflegenden Personen bin ich zufrieden	Stress

### Hypothesenformulierung

**Figure 3:**
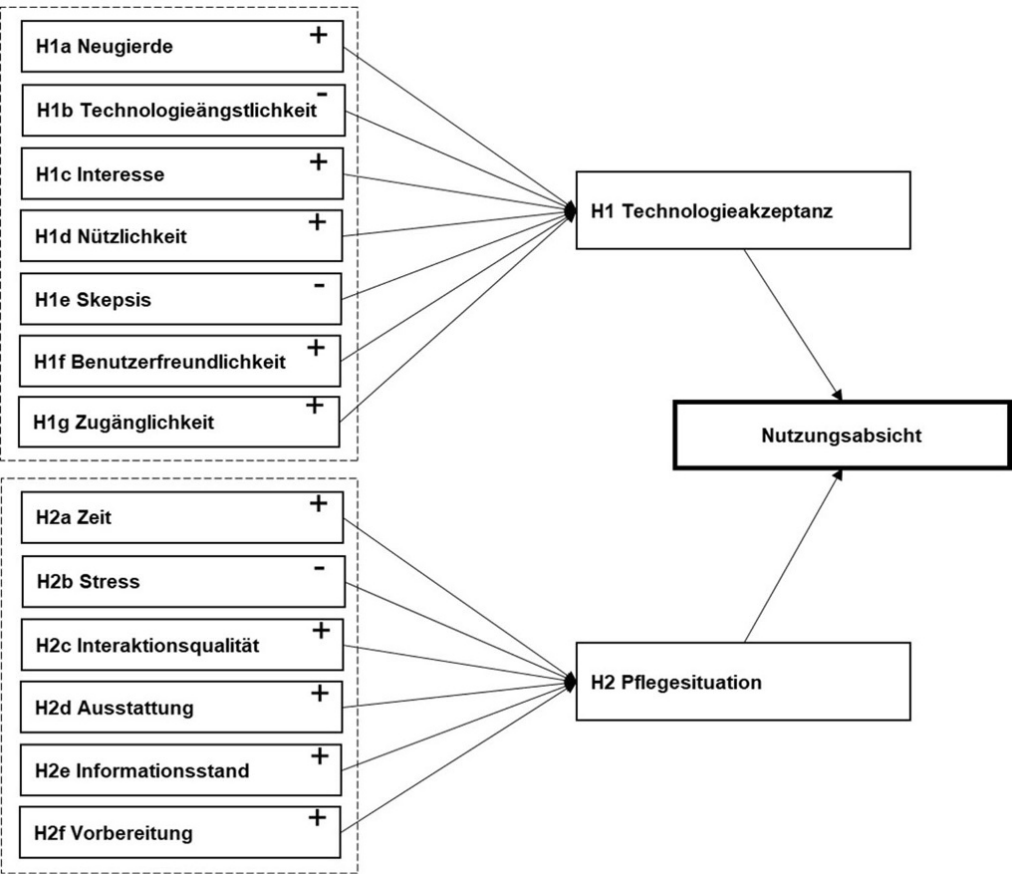

In einem vierten Schritt wurden auf Basis der Literaturanalyse und dem qualitativen Workshop Hypothesen zu den abgeleiteten Faktoren aufgestellt und die Wirkungsrichtungen in einem Modell zusammengefasst (vgl. Abb. 3). Zur Erklärung des vorgeschlagenen Modells, das die Faktoren zur Technologieakzeptanz sowie der am Fallbeispiel der Sensormatte erarbeiteten Faktoren zur Pflegesituation vereint, werden in den folgenden Tab. 3 und 4 die einzelnen gerichteten Hypothesen und ihre erwartete Wirkung auf die Nutzungsabsicht vorgestellt. Die Wirkungsrichtungen der Hypothesen orientieren sich dabei zum einen an den Technologieakzeptanzmodellen auf denen das vorgeschlagene Modell aufbaut und zum anderen an den durch die Pflegekräfte in Schritt 3 antizipierten Auswirkungen.

**Table 3:** 

Nr.	Hypothese	Erläuterung
H1	Technikakzeptanz hat einen positiven Effekt auf die Nutzungsabsicht der Pflegekräfte bezüglich der entwickelten Sensormatte	Den Grundannahmen des TUIs sowie des TAMs folgend resultiert aus einer hohen Technikakzeptanz auch eine Nutzungsabsicht (siehe Abschn. 2) (Davis 1989; Kothgassner et al. 2012)
H1a	Neugierde hat einen positiven Effekt auf die Technikakzeptanz der Pflegekräfte	Im TAM wird Neugierde als eine die Technikakzeptanz befördernde Eigenschaft eines Individuums bezogen auf den Gebrauch einer neuartigen Technologie beschrieben (Davis 1989). Eine vorhandene Neugierde, durch die das Individuum versucht herauszufinden, wie eine neue Technologie funktioniert, reduziert die empfundene Komplexität der Technologienutzung und unterstützt die Überwindung einer potenziellen Technologieängstlichkeit. Im Gegensatz dazu werden Individuen, die eine geringe Neugierde aufweisen, die neue Technologie ggf. als zu herausfordernd oder eine Auseinandersetzung mit ihr als langweilig empfinden (Kothgassner et al. 2012)
H1b	Technologieängstlichkeit hat einen negativen Effekt auf die Technikakzeptanz der Pflegekräfte	Technologieängstlichkeit beschreibt negative Gefühle oder Assoziationen bezüglich der Nutzung einer neuen Technologie. Sie äußert das Ausmaß der Bedenken oder Ängste des Individuums, wenn es mit der Möglichkeit konfrontiert wird, eine neue Technologie zu nutzen (Venkatesh und Davis 2000)
H1c	Interesse hat einen positiven Effekt auf die Technikakzeptanz der Pflegekräfte	Neben Neugierde und Technologieängstlichkeit inkludieren Kothgassner et al. (2012) im TUI zusätzlich den Faktor des grundsätzlichen Interesses des Individuums, sich mit einer neuen Technologie auseinanderzusetzen. Dabei wird erfasst, wie viel technisches Wissen ein Individuum generell aufweist und wie hoch die intrinsische Motivation ist, sich über den Fortschritt der Digitalisierung selbstständig zu informieren. Ein solches Interesse/Desinteresse spiegelt sich sowohl im privaten als auch im beruflichen Kontext in der Bereitschaft zur Techniknutzung wider
H1d	Nützlichkeit hat einen positiven Effekt auf die Technikakzeptanz der Pflegekräfte	Die Nützlichkeit einer Technologie ist definiert als das Ausmaß der Überzeugung eines Individuums, dass die Nutzung des neuen Systems die Ausführung der eigentlichen Aufgabe oder des Verhaltens verbessern wird (Davis 1989). Kothgassner et al. (2012) zeigen eine starke Korrelation zwischen der wahrgenommenen Nützlichkeit und der die Nutzungsabsicht bedingenden Technikakzeptanz
H1e	Skepsis hat einen negativen Effekt auf die Technikakzeptanz der Pflegekräfte	Skepsis wird als das Risiko oder die Unsicherheit beschrieben, die mit der Nutzung einer neuen Technologie verbunden ist. Auf einer individuellen Ebene geht es um den Zweifel, ob die betreffende Technologie bei der Erfüllung einer Aufgabe hilfreich ist oder nicht
H1f	Benutzer*innenfreundlichkeit hat einen positiven Effekt auf die Technikakzeptanz der Pflegekräfte	Venkatesh und Davis (2000) beschreiben Benutzer*innenfreundlichkeit als das Ausmaß, in dem eine Person glaubt, dass die Benutzung des Systems mühelos sein wird. Sie hat einen starken Einfluss auf die Nutzungsabsicht, da sie mit dem Stresserleben im Nutzungsprozess korreliert (Kothgassner et al. 2012)
H1g	Zugänglichkeit hat einen positiven Effekt auf die Technikakzeptanz der Pflegekräfte	Zugänglichkeit bezieht sich auf die Verfügbarkeit und Bequemlichkeit der Anschaffung einer neuen Technologie (Kothgassner et al. 2012). Im Kontext der ambulanten Pflege bedeutet dieser Faktor also, wie Pflegekräfte die Finanzierbarkeit und Beschaffung einer Technologie entweder seitens der Pflegedienste oder seitens der zu Pflegenden selbst einschätzen. Sofern dieser Anschaffungsprozess als kompliziert und nicht finanzierbar erachtet wird, reduziert dieser Faktor die Technikakzeptanz, wohingegen eine gute Zugänglichkeit sich als förderlich erwiesen hat (Kothgassner et al. 2012)

**Table 4:** 

Nr.	Hypothese	Erläuterung
H2	Eine erwartete Verbesserung der Pflegesituation hat einen positiven Effekt auf die Nutzungsabsicht der Pflegekräfte bezüglich der entwickelten Sensormatte	Aus den qualitativen Informationen des Workshops zur Pflegesituation wird abgeleitet, dass die erwartete Verbesserung der Pflegesituation einen positiven Einfluss auf die Nutzungsabsicht der digitalen Pflegetechnologie, also der betrachteten Sensormatte, hat
H2a	Ausreichend Zeit während der Pflege hat einen positiven Effekt auf die Qualität der aktuellen Pflegesituation	Durch den allgegenwärtigen Fachkräftemangel im Pflegesektor herrscht sowohl im stationären als auch im ambulanten Pflegebereich starker Zeitdruck für die Pflegearbeit. Eine Schicht im ambulanten Pflegedienst gestaltet sich durch eine abzufahrende Klient*innen-Route mit im Vorfeld festgelegten Zeitintervallen, die die Pflegekräfte bei den zu Pflegenden für die vereinbarten Leistungen zur Verfügung haben. Dabei sind die Zeitressourcen gesetzlich durch die Pflegezeitbemessung geregelt (Buchmann 2014). Ein Zeitverzug durch unerwartete Zwischenfälle im Pflegeplan wirkt sich negativ auf die Pflegesituation aus, da er sowohl für das Pflegepersonal als auch für die zu pflegenden Personen Stress erzeugt. Das aktuelle Zeitbudget und die erwartete Veränderung unter Nutzung einer neuen Technologie müssen auf faktorieller Ebene für die untersuchte Fragestellung also mitbetrachtet werden
H2b	Stress hat einen negativen Effekt auf die Qualität der aktuellen Pflegesituation	Zeitdruck ist nicht der einzige wiederkehrende, stresserzeugende Faktor für Pflegekräfte im ambulanten Dienst. Die Ungewissheit über den Gesundheitszustand der Gepflegten in der Abwesenheit des Pflegepersonals hat eine weitere maßbegliche Auswirkung auf das Stressempfinden der Pflegekraft. Durch die ausschließlich temporäre Betreuung von meist nur wenigen Minuten am Tag und eine eingeschränkte Vorbereitungsmöglichkeit können Sorgen beim Pflegepersonal vor oder nach dem Besuch bei den Gepflegten entstehen. Neue sensorbasierte Technologien können eine bessere Überwachung und Kontrolle des Gesundheitszustands der Klient*innen ermöglichen. In diesem Zusammenhang kann sowohl eine Stressreduktion durch eine Verringerung der Ungewissheit entstehen als auch eine Stresserhöhung durch die Option der durchgehenden Überwachung. Im Falle von Auffälligkeiten müsste von den Pflegekräften eine Reaktion initiiert werden, was wiederum den Personalbedarf und die Arbeitsbelastung erhöhen würde. Dieser Mechanismus wird im vorgeschlagenen Modell als Faktor „Stress“ beschrieben und ist losgelöst vom Zeitdruck als Faktor der psychischen Belastung des Pflegepersonals zu betrachten. Stressende Faktoren vermindern die Pflegequalität und verschlechtern somit die Pflegesituation
H2c	Eine gute Interaktionsqualität hat einen positiven Effekt auf die Qualität der aktuellen Pflegesituation	Die Interaktionsqualität umfasst neben der körperlichen Pflegearbeit auch das Ausmaß des persönlichen Gesprächs und Austauschs zwischen der Pflegeperson und den Klient*innen (Kumbruck 2010). Darüber hinaus gibt es im Pflegeberuf zusätzlich die Interaktion mit den Angehörigen der Gepflegten, welche häufig im selben Haushalt wohnen und im ambulanten Bereich meist aktiv in der Pflege integriert sind. Durch assistive Technologien, wie die hier beschriebene Sensormatte und die damit verbundene Automatisierung gewisser Pflegeprozesse, zum Beispiel die Messung und Dokumentation von Vitalwerten, bleibt dem Pflegepersonal nach der aktuellen Pflegezeitbemessung mehr Zeitbudget für die soziale Interaktion mit den Klient*innen und deren Angehörigen. Die Erhöhung des Zeitbudgets verbessert wiederum die Interaktionsqualität, die einen positiven Effekt auf die empfundene Qualität der Pflegesituation haben kann
H2d	Eine gute Ausstattung an verfügbaren Pflegehilfsmitteln hat einen positiven Effekt auf die Qualität der aktuellen Pflegesituation	Im Kontext ambulanter Pflege wird die „Ausstattung“ als verfügbare Pflegehilfsmittel, die den Pflegekräften für die Überwachung und Versorgung der Klient*innen zur Verfügung stehen, beschrieben. Die im Rahmen von DigiKomp-Ambulant entwickelte textile Sensormatte soll als ein solches digitales Hilfsmittel unterschiedliche Vitalparameter automatisiert aufzeichnen und über eine App an die Pflegekräfte zur Überwachung des Gesundheitszustands der Klient*innen vermitteln. Der Faktor der Güte der Ausstattung beschreibt die Ausstattung mit zur Verfügung stehenden Hilfsmittel für den Pflegeprozess bei den Gepflegten zuhause. Gelingt es durch die zur Verfügung stehenden Hilfsmitteln die pflegespezifischen Bedarfe der ambulanten Pflege zu adressieren, wird der Pflegeprozess vereinfacht. Hilfsmittel verbessern somit die aktuelle Pflegesituation
H2e	Ein guter Informationsstand über den Gesundheitsstatus der zu Pflegenden hat einen positiven Effekt auf die Qualität der aktuellen Pflegesituation	Der Informationsstand beschreibt den Kenntnisstand der Pflegekräfte von relevanten Ereignissen, wie zum Beispiel das Ausmaß der körperlichen Bewegung der Klient*innen während deren Abwesenheit. Wenn die Pflegetechnologie in der Lage ist, zusätzliche gesundheitsrelevante Informationen zu liefern, dann kann die Pflegekraft den Gesundheitszustand der Klient*innen besser verfolgen und die Pflegesituation zum Wohle der Klient*innen gestalten. Durch einen besseren Informationsstand kann die Pflege darüber hinaus prophylaktisch gestaltet und Spätfolgen, wie z. B. ein Dekubitus resultierend aus mangelnder Lagerung, vermieden werden
H2f	Das Gefühl auf den Zustand der zu Pflegenden vorbereitet zu sein hat einen positiven Effekt auf die Qualität der aktuellen Pflegesituation	Der Faktor Vorbereitung bezieht sich auf das Ausmaß, inwieweit die Pflegekräfte sich auf die individuellen Klient*innen und deren tagesaktuellen Bedürfnissen vorbereiten können. Da die Sensormatte das Potenzial hat die Vitalwerte der Klient*innen zu überwachen und aufzuzeichnen, kann die Vorbereitungsphase des nächsten Pflegetermins bereits anhand dieser Daten optimiert werden. Durch diese Möglichkeit zur gezielteren Vorbereitung kann der Grad an Unsicherheit für die Pflegekräfte bei Ankunft bei den zu Pflegenden reduziert werden

Der Struktur des Modells folgend werden zunächst die Faktoren vorgestellt, die basierend auf dem TUI die Technikakzeptanz erklären und beeinflussen (Modellstrang Technikakzeptanz, vgl. Tab. 3). Anschließend werden die Faktoren und zugehörigen Hypothesen erläutert, welche die Pflegesituation beeinflussen (Modellstrang Pflegesituation, vgl. Tab. 4).

## Methodik

Zur Evaluation des vorgeschlagenen Technikakzeptanzmodells wurden die fünfzehn Hypothesen (vgl. Tab. 3 und 4) mittels eines Online-Fragebogens getestet. Die Erhebung fand im Rahmen der ambulanten Pflege und vor der Technikeinführung statt. Da diese Studie den Zustand vor der Technikeinführung abbildet, wurden in der Befragung also die Erwartungen und Wünsche der Pflegekräfte an eine assistive Technologie zur Arbeitserleichterung erfasst. Die an das Fallbespiel angepassten 34 TUI-Items (siehe Tab. 1) wurden auf einer 7‑stufigen Likert-Skala von „Stimme überhaupt nicht zu“ bis „Stimme völlig zu“ abgefragt. Ergänzend dazu wurden die 35 qualitativ entwickelten Likert-Items (siehe Tab. 2) mit Bezug auf die Pflegesituation abgefragt. Neben den insgesamt 69 Items zur Operationalisierung der Nutzungsabsicht beinhaltet der Fragebogen zehn Items zur Erfassung relevanter Merkmale des demografischen Hintergrunds.

Der mit der Online-Software *Unipark* (Questback) programmierte Fragebogen erzielte in zwei Erhebungsphasen (September 2020 und Februar 2021) einen Datensatz von 45 vollständig durch Pflegekräfte ausgefüllte Fragebogen. Die Grundfunktionen der im hier vorliegenden Fallbeispiel untersuchten assistiven Technologie wurden zu Beginn des Fragebogens schriftlich erläutert, um einen einheitlichen Kontext für die Untersuchung der Technikakzeptanz zu schaffen.

In die statistische Analyse wurden nur vollständig ausgefüllte Fragebögen von Pflegekräften eingeschlossen, die bestätigt hatten, dass sie in der ambulanten Pflege tätig sind. Die Stichprobe ist charakterisiert durch ein Geschlechterverhältnis von null diversen, sieben männlichen und 38 weiblichen Versuchspersonen und einem Durchschnittsalter von 37 Jahren (*SD* = 13).

Zur statistischen Analyse der Daten wurde aus den beiden zur Verfügung stehenden Strukturgleichungsmodellansätzen PLS-SEM und CB-SEM (covariance based) der varianzbasierte Ansatz der Strukturgleichungsmodellierung (SEM), die sogenannte *Partial Least Square* (PLS) Methode, eingesetzt. Die Wahl des PLS-SEM Ansatzes beruht auf den folgenden Gründen:

- der PLS-SEM Ansatz ermöglicht ein exploratives Vorgehen. PLS-SEM strebt nicht danach, bereits etablierte Theorien zu untersuchen. Vielmehr ermöglicht PLS-SEM Zusammenhänge zwischen Konstrukten zu erforschen, die noch nicht fundiert untersucht wurden (Hair et al. 2019; Jahn 2007; Richter et al. 2016).
- PLS-SEM ist ein prognoseorientierter varianzbasierter Ansatz, der sich auf endogene Zielkonstrukte im Modell konzentriert. Er zielt darauf ab, deren erklärte Varianz (d. h. ihren R^2^-Wert) zu maximieren. Neben dem Aufdecken neuer Einflussfaktoren beschäftigt sich diese Arbeit mit einer Prognoserelevanz der eingebundenen, herausgearbeiteten Faktoren. Diese Fähigkeit wird insbesondere dem PLS-SEM Ansatz zugeschrieben (Hair et al. 2019; Jahn 2007).
- Der PLS-SEM Ansatz lässt sehr kleine, wie in dem vorliegenden Datensatz gegebene, Stichprobengrößen zu (Minimum 20) (Hair et al. 2019; Jahn 2007).
- Das aufgestellte Messmodell ist ein rein reflektives Messmodell mit einer hohen Anzahl von Faktoren. Für die strukturgleichungsbasierte Untersuchung mit vielen Faktoren wird die Nutzung unter zu Hilfenahme des varianzbasierten Ansatzes ausdrücklich empfohlen (Hair et al. 2019; Jahn 2007; Richter et al. 2016).

Für die Verarbeitung, Analyse und Interpretation der Daten und die Bewertung des Strukturmodells wurde die Software *SmartPLS* Version 3.3.3 verwendet.

## Ergebnisse

Das im Folgenden vorgestellte Auswertungsvorgehen orientiert sich an dem von Hair et al. (2017) postulierten generischen Vorgehen einer PLS-SEM Analyse. Es macht sich das von Hair et al. vorgestellte Vorgehen zu Nutze, während gleichzeitig ein auf den Forschungskontext angepasstes Vorgehen verfolgt wird. Im Folgenden werden die aussagekräftigsten Metriken und Messungen der PLS-SEM Methode (unter Berücksichtigung der genannten Gründe, insbesondere der Stichprobengröße) präsentiert, um die vorgestellten Hypothesen und das postulierte Modell zu testen und zu validieren.

Der gewählte Ansatz der PLS-SEM besitzt dabei, im Gegensatz zum CB-SEM kein universelles Maß des Modelfits (Goodness-of-Fit). In der Literatur gibt es zwar einen Vorschlag für ein globales Anpassungsgüte-Maß für PLS-SEM (Tenenhaus et al. 2004), bisherige Forschung zeigt allerdings, dass dieses Maß ungeeignet ist, um fehlerhafte oder fehlklassifizierte Modelle zu identifizieren (Marcoulides und Yuan 2017; Henseler und Sarstedt 2013). Die vorgeschlagenen Kriterien für ein universelles Modelfit-Maß befinden sich in einem frühen Stadium der Forschung, und sind deshalb (noch) nicht vollständig verstanden. In der PLS-SEM Analyse gilt deshalb der Wert von R^2^ als das wichtigste Kriterium für die Bewertung des Model-Fits. R^2^ hilft beispielsweise Einflüsse zu prognostizieren und beschreibt den Anteil der Varianz in der abhängigen Variable, der durch die unabhängige Variable erklärt werden kann. Infolgedessen nutzen viele Wissenschaftler*innen im PLS-SEM Ansatz die R^2^ Werte, die die Vorhersagefähigkeiten eines Modells anzeigen können und setzen dieses Kriterium gleichermaßen zur Qualitätsbeurteilung des Modells ein (Henseler und Sarstedt 2013).

In der folgenden Analyse handelt es sich um eine sorgfältige Auswahl der sinnvollsten und wertvollsten Kriterien zur Erklärung der Varianz in der abhängigen Variable durch die unabhängigen Variablen. Der PLS-SEM Ansatz konzentriert sich dabei auf die Diskrepanz zwischen den beobachteten oder angenäherten Werten der abhängigen Variablen und den durch das betreffende Modell vorhergesagten Werten (Hair et al. 2012a; b). Zur Bewertung der Qualität des Modells wurden die Werte zur internen Konsistenz durch Cronbachs Alpha und die Kongenerische Reliabilität (CR) berechnet. Für die Validität des Modells wurde die durchschnittlich erfasste Varianz (AVE) für alle Konstrukte im reflektiven Messmodell ausgewertet.

Außerdem wurde das Heterotrait-Monotrait-Kriterium (HTMT-Kriterium) für die Testung der diskriminanten Validität anstelle des Fornell-Larcker-Kriteriums und der Kreuzbelastungen betrachtet. Grund dafür ist eine empfindlichere Reaktion dieses Kriteriums auf Probleme mit der diskriminanten Validität. Ebenso stellt die Wahl des HTMT-Kriteriums den aktuellen Stand der Forschung in der PLS-SEM dar. Henseler et al. (2015) zeigen, dass das Fornell-Larcker-Kriterium und die Untersuchung von Kreuzbelastungen die fehlende diskriminante Validität in vielen Forschungssituationen nicht zuverlässig erkennen könnenn. Die Autoren schlagen daher einen alternativen Ansatz zur Beurteilung der diskriminanten Validität vor, der auf der Multitrait-Multimethoden-Matrix basiert: das Heterotrait-Monotrait-Verhältnis der Korrelationen. Henseler et al. (2015) demonstrieren die überlegene Leistungsfähigkeit dieses Ansatzes anhand einer Monte-Carlo-Simulationsstudie, in der sie den neuen Ansatz mit dem Fornell-Larcker-Kriterium und der Bewertung von (partiellen) Kreuzbelastungen vergleichen (Henseler et al. 2015).

### Bewertung der internen Konsistenz und diskriminanten Validität

Zur Überprüfung der internen Konsistenz des Modells wurde das Cronbachs Alpha berechnet. Dieser Maßzahl folgend ist die interne Konsistenz gewährleistet, wenn der Wert des Cronbachs Alpha mindestens 0,7 beträgt (Saunders et al. 2009). Alle Faktoren des aufgestellten Modells erfüllen dieses Kriterium. Um die interne Konsistenz des Modells mit einer weiteren Kennzahl, welche insbesondere dem Prinzip des PLS-SEM Ansatzes dient, zu unterstützen wurde zusätzlich die Kongenerische Reliabilität (CR) berechnet. Die für das vorliegende Modell errechneten CR-Werte überschreiten den empfohlenen Schwellenwert von 0,7 (Hair et al. 2016) für alle Faktoren (vgl. Tab. 5).

**Table 5:** 

Konstrukte	Cronbachs Alpha	CR	AVE
Nutzungsabsicht	0,846	0,752	0,649
Technikakzeptanz	0,758	0,797	0,612
Pflegesituation	0,711	0,719	0,551
Neugierde	0,836	0,891	0,671
Technologieängstlichkeit	0,729	0,753	0,642
Interesse	0,762	0,838	0,516
Nützlichkeit	0,874	0,914	0,727
Skepsis	0,703	0,746	0,576
Benutzerfreundlichkeit	0,774	0,868	0,688
Zugänglichkeit	0,743	0,829	0,749
Zeit	0,716	0,798	0,530
Stress	0,840	0,791	0,632
Interaktionsqualität	0,772	0,859	0,793
Ausstattung	0,780	0,856	0,548
Informationsstand	0,756	0,778	0,717
Vorbereitung	0,795	0,854	0,791

In der Bestimmung der Validität des aufgestellten Modells übersteigen die berechneten AVE-Werte den kritischen Schwellenwert von 0,5 (Fornell und Larcker 1981; Hair et al. 2014).

Um sicherzustellen, dass die aufgestellten reflektiven Konstrukte die stärksten Beziehungen zu den eigenen Indikatoren (im Vergleich zu jedem anderen Konstrukt) im PLS Modell haben, wurde die diskriminante Validität mit dem HTMT-Kriterium berechnet. Die Ergebnisse der Testung der diskriminanten Validität sind in Tab. 6 dargestellt. Alle Werte des HTMT-Kriteriums für die im Modell aufgestellten Konstrukte sind geringer als der empfohlene Schwellenwert von 0,85 (Kline 2015).

**Table 6:** 

Konstrukte	Ausstattung	Benutzerfreundlichkeit	Informationsstand	Intention to Use	Interaktionsqualität	Interesse	Neugierde	Nützlichkeit	Pflegesituation	Skepsis	Stress	Technologieakzeptanz	Technologieängstlichkeit	Vorbereitung	Zeit	Zugänglichkeit
Ausstattung	–	–	–	–	–	–	–	–	–	–	–	–	–	–	–	–
Benutzerfreundlichkeit	0,500	–	–	–	–	–	–	–	–	–	–	–	–	–	–	–
Informationsstand	0,598	0,491	–	–	–	–	–	–	–	–	–	–	–	–	–	–
Interaktionsqualität	0,816	0,634	0,734	–	–	–	–	–	–	–	–	–	–	–	–	–
Interesse	0,823	0,441	0,598	0,476	–	–	–	–	–	–	–	–	–	–	–	–
Neugierde	0,499	0,438	0,464	0,587	0,344	–	–	–	–	–	–	–	–	–	–	–
Nützlichkeit	0,685	0,561	0,516	0,844	0,291	0,615	–	–	–	–	–	–	–	–	–	–
Pflegesituation	0,777	0,699	0,630	0,816	0,475	0,490	0,818	–	–	–	–	–	–	–	–	–
Skepsis	0,490	0,489	0,611	0,670	0,579	0,465	0,480	0,641	–	–	–	–	–	–	–	–
Stress	0,714	0,726	0,527	0,747	0,518	0,329	0,588	0,829	0,586	–	–	–	–	–	–	–
Technologieakzeptanz	0,643	0,475	0,836	0,679	0,717	0,440	0,512	0,695	0,519	0,668	–	–	–	–	–	–
Technologieängstlichkeit	0,779	0,804	0,639	0,190	0,512	0,764	0,398	0,791	0,656	0,582	0,673	–	–	–	–	–
Vorbereitung	0,578	0,371	0,536	0,521	0,359	0,475	0,700	0,541	0,493	0,496	0,452	0,835	–	–	–	–
Vorbereitung	0,514	0,327	0,771	0,320	0,645	0,454	0,258	0,351	0,849	0,284	0,476	0,405	0,361	–	–	–
Zeit	0,623	0,226	0,566	0,361	0,727	0,214	0,165	0,324	0,839	0,198	0,529	0,297	0,229	0,697	–	–
Zugänglichkeit	0,510	0,468	0,363	0,665	0,394	0,412	0,386	0,744	0,453	0,611	0,444	0,842	0,590	0,240	0,265	–

### Auswertung des postulierten Modells

Um das vorgeschlagene Strukturgleichungsmodell zu evaluieren, wurden die Beziehungen zwischen den verschiedenen Konstrukten innerhalb des Modells analysiert. Dieses Verfahren beinhaltete das Testen der Qualität der kausalen Beziehungen zwischen allen latenten Variablen und die Identifizierung des relativen Gewichts jeder der unabhängigen Variablen innerhalb des Modells. Zu diesem Zweck wurde das Bootstrapping-Verfahren mit 2000 Unterstichproben durchgeführt (vgl. Xu et al. 2020). Die Hypothesentestung erfolgt durch die Analyse der standardisierten Pfadkoeffizienten zwischen den Konstrukten in Ergänzung zu den entsprechenden *p*-Werten und *t*-Werten, die durch das Bootstrapping-Verfahren berechnet wurden. Die Pfadkoeffizienten geben die relative Stärke des Einflusses der unabhängigen Variablen auf die abhängigen Variablen an.

Alle angegebenen Hypothesen im vorgeschlagenen Strukturmodell erweisen sich auf einem Signifikanzniveau von 0,05 für den *p*-Wert und 1,96 für den *t*-Wert als signifikant. Eine Übersicht über die Pfadkoeffizienten und die genannten statistischen Maße ist in Tab. 7 dargestellt.

**Table 7:** 

Hypothese	Beziehung	Pfadkoeffizient	*t*-Wert	*p*-Wert
H1	Technikakzeptanz (+^a^) → Intention to Use	0,761	3,566	0,012
H2	Pflegesituation (+) → Intention to Use	0,152	3,331	0,046
H1a	Neugierde (+) → Technikakzeptanz	0,211	3,526	0,043
H1b	Technologieängstlichkeit (−^a^) → Technikakzeptanz	−0,124	2,061	0,026
H1c	Interesse (+) → Technikakzeptanz	0,153	3,270	0,008
H1d	Nützlichkeit (+) → Technikakzeptanz	0,332	3,517	0,035
H1e	Skepsis (−) → Technikakzeptanz	−0,174	2,193	0,014
H1f	Benutzerfreundlichkeit (+) → Technikakzeptanz	0,167	3,347	0,019
H1g	Zugänglichkeit (+) → Technikakzeptanz	0,165	3,484	0,038
H2a	Zeit (+) → Pflegesituation	0,124	3,282	0,033
H2b	Stress (−) → Pflegesituation	−0,241	2,337	0,020
H2c	Interaktionsqualität (+) → Pflegesituation	0,292	3,488	0,005
H2d	Ausstattung (+) → Pflegesituation	0,195	3,310	0,027
H2e	Informationsstand (+) → Pflegesituation	0,236	3,485	0,021
H2f	Vorbereitung (+) → Pflegesituation	0,247	3,479	0,017

^a^(+) → positiver Zusammenhang, (−) → negativer Zusammenhang

Um das Vorhandensein von Multikollinearität zwischen den Indikatoren auszuschließen, wurden die Werte des Varianzinflationsfaktors (VIF) gemessen. Im Modell sind alle VIF-Werte kleiner als 3,3, sodass keine signifikante Multikollinearität vorliegt (Wetzels et al. 2009). Eine Übersicht über die VIF-Werte der unabhängigen Variablen ist in Tab. 8 dargestellt.

**Table 8:** 

Konstrukte	Nutzungsabsicht	Pflegesituation	Technikakzeptanz
Ausstattung	–	1,859	–
Benutzerfreundlichkeit	–	–	1,741
Informationsstand	–	2,820	–
Interaktionsqualität	–	2,391	–
Interesse	–	–	1,724
Neugierde	–	–	1,965
Nützlichkeit	–	–	1,733
Pflegesituation	1,246	–	–
Skepsis	–	–	2,591
Stress	–	1,950	–
Technikakzeptanz	1,261	–	–
Technologieängstlichkeit	–	–	1,847
Vorbereitung	–	1,913	–
Zeit	–	1,667	–
Zugänglichkeit	–	–	1,962

Zur Überprüfung, ob die beobachteten statistischen Effekte sinnvoll die zu erklärenden Variablen erklären bzw. die aufgestellten Indikatoren eine praktische Relevanz aufweisen wurde die Effektgrößen (f^2^) für das aufgestellte Modell berechnet (siehe Tab. 9; Hair et al. 2014; Kock und Hadaya 2018). Um einen Effekt zu beobachten, muss die Effektgröße mindestens über 0,02 für alle Variablen liegen. Bei einer Effektgröße ab 0,15 wird von einem mittleren Einfluss und bei einer Effektgröße ab 0,35 von einem starken Einfluss gesprochen (Hair et al. 2014).

**Table 9:** 

Konstrukte	Nutzungsabsicht	Pflegesituation	Technikakzeptanz
Ausstattung	–	0,268	–
Benutzerfreundlichkeit	–	–	0,263
Informationsstand	–	0,274	–
Interaktionsqualität	–	0,289	–
Interesse	–	–	0,252
Neugierde	–	–	0,311
Nützlichkeit	–	–	0,394
Pflegesituation	0,261	–	–
Skepsis	–	–	0,377
Stress	–	0,193	–
Technikakzeptanz	0,427	–	–
Technologieängstlichkeit	–	–	0,365
Vorbereitung	–	0,224	–
Zeit	–	0,215	–
Zugänglichkeit	–	–	0,259

Alle Effektgrößen des aufgestellten Modells befinden sich über einem Wert von 0,15. Damit zeigt sich ein moderater Einfluss der Variablen auf die zu erklärenden Variablen Nutzungsabsicht, Pflegesituation und Technikakzeptanz. Für die Einflüsse von Neugierde, Nützlichkeit, Skepsis und Technologieängstlichkeit auf die Technikakzeptanz, sowie der Einfluss von Technikakzeptanz auf die Nutzungsabsicht kann sogar ein großer Einfluss festgestellt werden. Etwas schwächer im Vergleich zu den anderen Effektstärken zeigt sich der Einfluss von der Variable Stress auf die Pflegesituation. Mit einem Wert von 0,193 zeigt sich der Effekt jedoch immer noch als hinreichend um einen Einfluss von Stress auf die Pflegesituation beobachten zu können.

Der Modell-Fit des vorgeschlagenen Modells wird durch die Betrachtung der R^2^- und R^2^-adjustierten Werte der Hauptkonstrukte abgebildet, die den Betrag der erklärten Varianz durch die einzelnen Faktoren beschreiben. Das vorgeschlagene Technikakzeptanzmodell für die ambulante Pflege erklärt zwischen 21,8 % (R^2^ adj.) und 44,9 % der Varianz in der Verhaltensabsicht die im Fallbeispiel betrachtete sensorbasierte Pflegetechnologie zu nutzen. Zur Begründung der Einflüsse auf die Technikakzeptanz erklärt das aufgestellte Modell zwischen 18,2 % (R^2^ adj.) und 31,2 % der Varianz in der Technikakzeptanz. Außerdem kann das Modell zwischen 13,2 % (R^2^ adj.) und 25,1 % der Varianz des Faktors Pflegesituation (vgl. Tab. 10) erklären.

**Table 10:** 

Konstrukte	R^2^	R^2^ (adjustiert)
Nutzungsabsicht	0,449	0,218
Technikakzeptanz	0,312	0,182
Pflegesituation	0,251	0,132

Die Einschätzung der Schwelle, ab der der R^2^-Wert als erklärungsstark angesehen wird, steht in starker Abhängigkeit der Disziplin. Falk und Miller (1992) empfehlen, dass R^2^-Werte disziplinunabhängig mindestens gleich oder größer als 0,10 sein sollten, damit die Varianzerklärung eines bestimmten endogenen Konstrukts als angemessen angesehen werden kann. Cohen (1988) schlägt vor, dass R^2^-Werte für endogene latente Variablen wie folgt bewertet werden: 0,26 (substantiell), 0,13 (moderat), 0,02 (schwach). Daher wird die Aussagekraft der statistischen Ergebnisse des Modells für ausreichend stark als Ausgangsmodell erachtet.

## Diskussion

### Theoretische und praktische Implikationen

Mit der SEM-PLS-Analyse können sowohl die beiden Haupthypothesen H1 und H2 als auch alle dreizehn Teilhypothesen statistisch bestätigt werden. Das Forschungsmodell zur Beschreibung von beeinflussenden Faktoren für die Ausbildung der Nutzungsintention der textilen Sensormatte in der ambulanten Pflege vor der Technikeinführung zeigt, dass die Konstrukte Technikakzeptanz und Pflegesituation die Varianz der abhängigen Variablen „Nutzungsabsicht der im Fallbeispiel untersuchten sensorischen Textilmatte“ zu 44,9 % (R^2^) bzw. 21,8 % (R^2^ adjustiert) aufklären können (H1).

Die Ergebnisse zeigen zudem, dass die Faktoren Neugierde, Technologieängstlichkeit, Interesse, Nützlichkeit, Skepsis, Benutzerfreundlichkeit und Zugänglichkeit die Varianz der Variable „Technikakzeptanz der Sensormatte“ zwischen 18,2 % (R^2^ adj.) und 32,1 % aufklären können. Genauer zeigte sich, dass Neugierde (H1a), Technologieängstlichkeit (H1b), Interesse (H1c), Nützlichkeit (H1d), Skepsis (H1e) Benutzerfreundlichkeit (H1f) sowie Zugänglichkeit (H1g) statistisch signifikante Prädiktoren der Technikakzeptanz der untersuchten Pflegetechnologie-Nutzung sind. Unter diesen zeigt der Faktor Nützlichkeit (H1d) den stärksten Effekt auf die Technikakzeptanz. Dieses Ergebnis ist bei der Entwicklung von Technologien für die professionelle Pflege von besonderer Relevanz, da dieser Effekt signalisiert, dass die Nützlichkeit einer bestimmten Technologie besonders im ambulanten Pflegekontext elementar ist. Die Nützlichkeit der Technologie ist hier in Abgrenzung zur Pflegesituation zu verstehen. Die Nützlichkeit beschreibt dabei, wie nützlich die untersuchte Technologie ist, die in der Pflegesituation anfallenden Aufgaben zu bewältigen. Die Pflegesituation wird wiederum durch die Wahrnehmung von Stress, Informationsstand, Ausstattung, Zeit, Vorbereitung und Interaktionsqualität bestimmt.

Häufig wird die Nützlichkeit einer Pflegetechnologie für die Anwendung in einer ambulanten Pflegesituation von den Technikentwickler*innen nicht ausreichend adressiert. Dies findet in einem unzureichenden Verständnis pflegespezifischer Bedarfe seinen Ursprung (Weinberger und Decker 2015). Als eine Ursache hierfür wird oft die Sprachbarriere zwischen Technikentwickler*innen und Technikanwender*innen in der Pflege gesehen, die eine nutzerorientierte Technikentwicklung behindert.

Ein weiterer Einfluss auf die Technikakzeptanz zeigt sich durch den Effekt der Zugänglichkeit zur Technologie (H1g). Dieser durch das vorliegende Modell statistisch bestätigte Einfluss für den Einsatz einer Pflegetechnologie in der ambulanten Pflege deckt sich mit Befunden vorheriger Forschung zur Nutzungsabsicht von neuen Technologien in der Pflege. Hier wird herausgestellt, dass Technologien zwar häufig bekannt, jedoch für die Pflegekräfte in ihrem Arbeitsalltag nicht verfügbar sind (Kuhlmey et al. 2019). Die Ursache hierfür liegt meist in einer problematischen finanziellen Anschaffungssituation für ambulante Pflegedienste oder seitens der Klient*innen. In der vorliegen Studie ist es überraschend, dass dem Faktor Zugänglichkeit bereits in der Untersuchung von Einflussfaktoren vor der Technikeinführung ein solch hoher Stellenwert zukommt. Dieser Faktor ist als organisationaler Faktor insbesondere für Pflegedienste und Technikentwickler*innen relevant, da die Zugänglichkeit zu einer Technologie einen Faktor darstellt, den der Pflegedienst oder Technikhersteller*innen maßgeblich beeinflussen können, die Pflegekraft allerdings nicht. Für die Praxis lässt sich daraus ableiten, dass neben der inhaltlichen Technikentwicklung auch die Finanzierung bzw. die Geschäftsmodelle für ambulante Pflegetechniken frühzeitig im Einführungsprozess vorangetrieben werden müssen um eine Zugänglichkeit und damit eine Nutzung der Technologie zu ermöglichen.

Bezogen auf das Konstrukt der erwarteten Veränderung der Pflegesituation durch den Einsatz von Technik kann im Rahmen dieser auf das vorliegende Fallbeispiel bezogenen Studie erstmals gezeigt werden, dass die untersuchten berufsspezifischen erwarteten Faktoren (Zeit, Stress, Interaktionsqualität, Ausstattung, Informationsstand und Vorbereitung) den Anteil der Varianz der abhängigen Variable „Pflegesituation“ zwischen 13,2 % (R^2^ adj.) und 25,1 % aufklären können. Da es sich hier um eine Erhebung vor Technikeinführung handelt, sind die Faktoren als erwartete Veränderungen zu verstehen.

Im Einzelnen zeigt sich, dass erwartete Interaktionsqualität (H2c), erwartete Vorbereitung (H2f) und erwarteter Informationsstand (H2e) die signifikantesten Prädiktoren für die Pflegesituation bei der erwarteten Nutzung der textilen Sensormatte sind. Die praktische Relevanz dieser drei Faktoren in Ihrem erwarteten Einfluss auf die Pflegesituation wird auch durch die Effektgrößen (f^2^) unterstrichen. Für den Faktor Ausstattung zeigt sich zwar ein eher geringer Pfadkoeffizient, die Effektgröße dieses Faktors drückt jedoch aus, dass die Ausstattung einen statistisch relevanten Einfluss auf die Pflegesituation hat.

Die untersuchten Faktoren Interaktionsqualität und Vorbereitung beeinflussen die erwartete Pflegesituation am stärksten. Die Stärke dieser Effekte unterstreichen die Relevanz der sozialen und emotionalen Facetten des Pflegeberufs, deren Wirkung auf Pflegesituationen und schließlich auch auf die Nutzungsabsicht von Technologienutzung im ambulanten Pflegebereich.

In der Zukunft werden neuen Technologien einen Teil der Pflegeprozesse nach Möglichkeit automatisieren. Teile des im Rahmen dieser Arbeit entwickelten Faktors Interaktionsqualität werden durch die Variablen „bisher zur Verfügung stehende Zeit der Pflegearbeit“ und der „Erwartung an die zur Verfügung stehende Zeit der Interaktion mit der zu Pflegenden Person durch den Einsatz von Technik“ beschrieben. Der statistisch untersuchte positive Einfluss der Interaktionsqualität auf die Pflegesituation drückt damit auch aus, dass mehr Zeit in der Pflege für die Interaktion mit den zu Pflegenden gewünscht ist und dies als Resultat des Einsatzes von Technik erwartet wird.

In bisheriger Forschung zeigt sich, dass vor allem die psychische Arbeitsbelastung durch eine bessere Möglichkeit zur Vorbereitung auf die vorzufindende Situation bei den zu Pflegenden mittels Einsatzes von Technologie reduziert werden kann (Hülsken-Giesler et al. 2019). Der im Rahmen dieser Studie herausgestellte statistisch signifikante Einfluss der Variable Stress und ihre Wirkung auf den Faktor Pflegesituation unterstützt dieses Ergebnis und zeigt deutlich, dass erwarteter Stress einen Einfluss auf die erwartete Pflegesituation nimmt.

Ebenfalls damit einher geht der positive Einfluss der hier untersuchten Variable des Informationsstandes. Im Rahmen dieser Variable wurde abgefragt ob die Option, Informationen über Bewegungen und Vitaldaten zu liefern, wenn die Pflegekräfte nicht vor Ort sind, einen positiven oder negativen Einfluss auf das Empfinden der Pflegesituation verursacht. Die Versorgung mit ausreichend Informationen zeigt dabei einen positiven Effekt auf die erwartete Pflegesituation. Im Modell zeigen die Faktoren „Vorbereitung“ und „Informationsstand“ einen signifikanten Einfluss auf die erwartete Pflegesituation. Die in dieser Studie eingesetzte Pflegetechnologie fungiert als Informationssystem, welches automatisiert Pflegeinformationen über das Zeitintervall der eigentlichen Pflege hinaus bereitstellen kann. Besonders die Informationen über das Ausmaß von Bewegungen bettlägeriger Klient*innen kann beispielsweise zur besseren Dekubitusprophylaxe genutzt werden und somit langfristig den Pflegeaufwand durch Folgeerscheinungen reduzieren (Schröder 2015).

In der qualitativen Ableitung der Items zur Pflegesituation wurde zudem herausgestellt, dass die Möglichkeit zur dauerhaften Überwachung von Klient*innenzuständen ein hohes Stresspotential erzeugen kann. Dieser vermutete negative Einfluss von Stress auf die Pflegesituation kann auch im aufgestellten Modell statistisch bestätigt werden (H2b).

Die vorgestellten R^2^-Werte für die Nutzungsabsicht, die dafür notwendige Technikakzeptanz und der Einfluss der aktuellen Pflegesituation bilden nach Cohen (1988) moderate Effektstärken ab. Das am Fallbeispiel vorgestellte Forschungsmodell zur Technikakzeptanz einer Sensormatte in der ambulanten Pflege kann also nicht den vollständigen Anteil der Varianz der abhängigen Variable „Nutzungsabsicht“ durch die vorgestellten unabhängigen Variablen aufklären, obgleich die Anpassung des TUIs (Kothgassner et al. 2012) an den Kontext der ambulanten Pflege und die Erweiterung der Faktorenebene der Pflegesituation dazu beigetragen haben Unterschiede in der Nutzungsabsicht von Pflegetechnologien ambulanter Pflegekräfte professionsspezifisch zu erklären. Dies führt zu der Annahme, dass die Nutzungsabsicht von assistiven Pflegetechnologien im ambulanten Anwendungsbereich in Wirklichkeit von noch mehr Faktoren aufgeklärt wird, als die, die bei der vorliegenden Erhebung mittels Online-Fragebogen tatsächlich gemessen wurden.

Die vorliegende Studie hatte zum Ziel Faktoren herauszuarbeiten, die die Nutzungsabsicht von Pflegekräften in Bezug auf neue Technologien beeinflussen, um daraus praxisrelevante Orientierungspunkte für die partizipative Technikentwicklung ableiten zu können. Das vorgestellte Modell konnte dabei erstmals zeigen, dass neben klassischen Faktoren zur Technologieakzeptanz die integrierte, professionsspezifische Ebene der Pflegesituation bei zukünftiger Forschung im Bereich der Nutzungsabsicht von Technologie in der Pflege obligatorisch inkludiert werden sollte.

### Limitationen der Studie

Das pflegespezifische Technikakzeptanzmodell, welches sich bislang aus dem beschriebenen Forschungsprojekt entwickelt hat, ist aufgrund einer für die PLS-SEM-Analyse recht kleinen Stichprobe (*N* = 45) als erste fallbezogene Pilotierung zu betrachten. Dies bedeutet, dass die Untersuchung erste Erklärungsansätze aufzeigt, das Modell jedoch mittels zukünftiger Forschung durch weitere varianzaufklärende Faktoren ergänzt werden muss, um den Anteil der aufklärenden Varianz zu erhöhen. Ein Grund für die geringe Stichprobengröße ist der Fokus auf Mitarbeitende von zwei im Projekt DigiKomp-Ambulant beteiligten Pflegediensten. Durch den Einbezug weiterer Pflegedienste außerhalb des zugrundeliegenden Projektes könnte die Stichprobe vergrößert und so die Aussagekraft des Modells gestärkt werden. Weitere Untersuchungen zur betrachteten Sensormatte sowie weitere Fallstudien können außerdem dazu beitragen, aus dem fallbezogenen Modell belastbare Rückschlüsse auf die Nutzungsabsicht in Bezug auf digitale Pflegetechnologien im Allgemeinen zu ziehen.

Es handelt sich bei der vorliegenden Studie um eine Untersuchung vor Technikeinführung. Die durchgeführte Online-Umfrage kann demnach nur die erwartete Nutzungsabsicht darstellen. Zur Überprüfung der tatsächlichen Nutzungsabsicht nach Technikeinführung ist eine weitere Umfrage vorzunehmen. Das aufgestellte Modell kann dennoch bereits jetzt aufzeigen, dass die Berücksichtigung klassischer Technikakzeptanzfaktoren in der Technikentwicklung verpflichtend ist. Ebenfalls zeigt das Modell auch statistisch die Wichtigkeit der Inklusion der Pflegesituation in der Nutzungsintentionsbewertung.

Bei Technikakzeptanzuntersuchungen im beruflichen Kontext sind soziotechnische Umgebungseinflüsse und dadurch entstehende Limitationen neben der eigentlichen Forschungsfrage zu berücksichtigen. Der Ausbruch und die Ausbreitung der SARS-CoV-2-Pandemie hat den Datenerhebungsprozess der Studie stark beeinflusst. Die Pandemie-Situation verschlechterte sich während des Forschungs- und Untersuchungszeitraums, sodass das Pflegepersonal in allen Bereichen des deutschen Gesundheitssystems einer extrem hohen Arbeitsbelastung ausgesetzt war (Hower et al. 2020). Diese nicht-kontrollierbaren Rahmenbedingungen müssen bei der Einordnung der Repräsentativität sowie der Dauergültigkeit der Ergebnisse berücksichtigt werden. Bedingt durch die SARS-CoV-2-Pandemie gestaltete sich die Rekrutierung von partizipierenden ambulanten Pflegediensten zudem als schwierig, was sich wiederum negativ auf die Stichprobengröße auswirkte.

### Fazit und Forschungsausblick

Die Nutzungsintention von Technologien in der ambulanten Pflege ist ein umfassendes Konstrukt, dem eine Vielzahl an erklärenden Faktoren zugrunde liegt. Durch die Überprüfung der Faktoren zur Technikakzeptanz sowie dem erstmaligen Einbezug beeinflussender Faktoren der Pflegesituation konnte in Bezug auf das betrachtete Fallbeispiel gezeigt werden, dass die Pflegesituation einen erheblichen Einfluss auf die Nutzungsintention der Pflegekräfte hat. Ein Ausbleiben des aktiven Einbezugs von Pflegekräften in die Technikentwicklung kann also in einer nicht ausreichenden Adressierung berufsspezifischer Bedarfe und schließlich in einer mangelnden Nutzungsabsicht resultieren.

Für erfolgreiche Technikentwicklungen ist es daher entscheidend, ein Verständnis für die notwendigen Eigenschaften von Pflegetechnologien zu entwickeln und die Determinanten zu identifizieren, die eine professionsspezifische, hohe Technikakzeptanz sowie eine Verbesserung der aktuellen Pflegesituation begünstigen. Das entwickelte Strukturgleichungsmodell legt durch die Beschreibung von begünstigenden und hemmenden Faktoren der Nutzungsintention von Pflegekräften im ambulanten Pflegedienst vor der Technikeinführung einen wichtigen Grundstein in der pflegespezifischen Arbeitswissenschaft. Damit trägt das beschriebene Fallbeispiel aus dem Forschungsprojekt DigiKomp-Ambulant zum Verständnis bei, welche Anforderungen und Bedürfnisse die spätere Nutzungsabsicht für Pflegetechnologien prägen und somit die Arbeitsbedingungen in diesem Berufssektor verbessern können.

Ein kausaler Zusammenhang zwischen den Faktoren Technologieakzeptanz und Pflegesituation wurde im vorliegenden Modell nicht untersucht. In der Entwicklung des Modells ergaben sich hierfür in der Literatur keine Hinweise für einen Zusammenhang. Vielmehr war es Ziel dieser Studie die einzelnen Einflüsse der beiden Faktoren Technikakzeptanz und Pflegesituation auf die Nutzungsabsicht der textilen Sensormatte herauszuarbeiten, um so praxisrelevante Akzeptanzfaktoren für die Nutzer*innengerechte Technikentwicklung ableiten zu können.

Zum Verständnis von Handlungen und Einstellungen bezüglich der Technikakzeptanz von Pflegekräften bietet es sich jedoch für zukünftige Forschung an, den Zusammenhang zwischen der Bewertung einer Pflegesituation und der daraus resultierenden Technologieakzeptanz näher zu untersuchen. Die positive oder negative Bewertung einer Pflegesituation könnte dabei einen starken Einfluss auf die Akzeptanz von neuer Technik ergeben. Ebenfalls ist es möglich, durch Re-modellierung des Modells eine aus der Pflegesituation folgende Technikakzeptanzabschätzung zu untersuchen.

Im weiteren Projektverlauf des DigiKomp-Ambulant-Projekts ist eine Einführung der sensorbasierten Pflegetechnologie bei den kooperierenden ambulanten Pflegediensten geplant. Vor und nach dieser Einführung werden Pflegekräfte mithilfe eines teilstrukturierten Interviews zur Thematik der Technikakzeptanz und der Nutzungsabsicht befragt. Resultierend daraus kann das hier aufgestellte Modell durch qualitative Daten sowohl tiefergehend kausal erklärt, als auch induktiv durch zusätzliche relevante Faktoren erweitert werden.

Über den Rahmen des Forschungsprojekts mit direktem Bezug zur entwickelten textilen Sensormatte hinaus, ist die Übertragbarkeit des untersuchten Modells auf die Einführung weiterer Pflegetechnologien im ambulanten Dienst zu erforschen. Dies ist insbesondere deshalb von Bedeutung, da die Ergebnisse der vorliegenden Studie aufgrund der fallbezogenen Betrachtung, Modellentwicklung und -validierung nicht den Anspruch einer Verallgemeinerung erfüllen können. Sie bilden jedoch eine Grundlage und geben wichtige Ansatzpunkte für weitere Studien und Fallbeispiele, mit deren Hilfe ein allgemeines Technikakzeptanzmodell für Pflegetechnologien abgeleitet werden kann. Ein solches Modell kann nicht zuletzt zu einer nutzer*innenzentrierten Entwicklung und Einführung digitaler Pflegetechnologien und zur Unterstützung von Pflegenden beitragen.
